# Cremation

**Published:** 1879-10

**Authors:** 


### CREMATION.

What follows was written for the *Sunday Telegram*, of this city, and in looking it over, can not see that we can do better than reproduce it in our own columns. That our present burial system must and will be abandoned as our cities grow larger and our people wiser, we do not doubt. Every one must see the danger that lurks in the decaying body, and, when a few more households have been stricken with diptheria, typhoid fever, and other infective and contagious diseases, sensible people will be willing to part with a mere sentimentality about the disposition of the dead, and will at last look upon cremation as the most cleanly and beautiful ceremony yet devised for disposing of the dead.

But, here is the article :—

Fewer changes have occurred in our burial system than in any other ceremony practiced and recognized by civilized nations. This is due probably to our ideas of reverence for the dead and a shrinking from practicing any ceremony in disposing of them differing from that observed by our ancestors. Notwithstanding, some innovations have occurred that seem to have been brought about more as a means of comfort and healthfulness to the living, than consideration for the dead.

The Egyptians, somewhere in the sixth century, possessed a happy faculty of converting their dead into mummies, until they became so numerous as to hinder the living. The Greeks profiting by this experience, placed their dead bodies in coffins constructed of baked clay or porcelain and buried them outside the city limits that no pollution might come to the living, thus early recognizing the danger to health from decomposing animal matter.

To the Greeks are we also indebted for the next change,—the cleanliest and most wholesome of all—cremation; this they practiced early in the same century. The bodies were placed upon pyres of light inflammable wood and burned until nothing but the bones remained. These were collected, placed in fancifully wrought urns that were deposited in niches in tombs constructed by the roadsides, just outside the city walls.

The Romans, we are told, buried their dead, without any embalming or preservative preparation, and left them to rot, as we do to-day; although, in the early days of the Republic they became wiser and often practiced cremation. All their burials occurred outside the city walls, however, nothing of the sort being permitted near the habitations of the living.

The early Christians are responsible for the church yard burial grounds, many of which are still to be found in our older cities and in all country towns and villages.

As people became wiser and more familiar with the laws of health, these churchyard burial places gave way for the cemeteries that were located away from the densely populated districts.

But now we begin to be aware of the danger to public health from our cemeteries, brought about by the following recognized facts :—

Cemeteries are usually located upon hills, but just why, is past finding out, unless it be to favor the deleterious consequences emanating therefrom.

The burial place being high, is usually dry, thus favoring slow rotting of the body. The dry earth absorbs the products of decomposition until through a multiplicity of bodies within a small area, it can do so no longer, then the superabundant nastiness is washed by the rains into the underground water courses, to find its way into the wells in the vicinity.

It has been found that in Woodlawn cemetery in this city, from eight to twelve years are necessary to completely rot and dispose of the fleshy substance of an adult body. Imagine the vile accumulation of rottenness and pollution existing there from the accumulated dead during such a period!

That the percolation of this highly poisonous matter into our water courses produces dangerous and fatal sickness, we need not stop to argue. Any physician will so inform you. That it caused the terrible epidemic of diphtheria, from which this city suffered last winter, is not unlikely. While physicians do not entirely agree as to the cause of diphtheria, they are in perfect accord in declaring it to be most prevalent and malignant in localities where filth abounds or where drinking water is contaminated by cess-pools, privies, cemeteries, or stagnant and filthy water of any sort.

In the epidemic referred to, it was known, from the published statistics of deaths, that the largest number of fatal diphtheritic cases occurred in the Sixth ward. This ward is located at the foot of our principal cemetery. The circumstance may have no significance, but you will find but few, if any, medical men that do not consider it quite likely that the contagion had its origin in the wells that received a poisonous taint from the cemetery as well as the adjacent canal.

This may seem a somewhat circuitous route to reach the point aimed at in the begining of this article, but leads us to enquire: is it not strange that, having advanced and improved in all other habits and customs of life, we should still adhere to the pernicious method of burying our dead? Queer, is it not, that we should be willing to subject ourselves and children to the dangerous and deadly influences of putrid, decaying bodies, and that we suffer our dead, who were so dear to us in life, to be in a loathsome grave, festering in nastiness, while horrid beetles and slimy worms feast upon the putrid body, wiggle through its ribs, the gaping mouth and vacant orbs, and hide within the skull. Is it not horrible to look upon the stinking, slimy mass, with blackened bones, and demoniacal, grinning features, when disinterred for burial in some more favored spot? Can you bear to think of the body of your sweet, pure child thus slowly rotting away, a hiding and feasting place for worms and bugs, when a cleanly, pure, beautiful method of disposing of the body is offered in *cremation?*

Fire is the poetry of cleanliness and purity. To prepare the body as loving hands are wont to, placing it upon a bright platinum sheet, then, while our trusted pastor offers a prayer and we bow our heads in supplication to the Divine Master for the repose of the soul of our loved one, friendly loving hands gently glide the wicker casket within the burning tomb, the bright, pure fire envelops it, the door closes, and we turn away thankful that no disgusting rottenness can pollute that loved form. In two hours, the white and cleanly ashes of the purified body are brought you, which are deposited in the earth, over which the costly shaft or modest tablet may rest; or are placed in an urn, above the ground, to be garlanded with flowers in your visitation to the sacred spot. Then, when you look upon its resting place, what a sense of satisfaction must come to you, knowing that the body of your loved one is as pure and undefiled as you would have had it in life, with no possibility of its poisoning and sickening the living who wait and mourn.

---

In order to show that this subject is being considered elsewhere, we copy the following from the Philadelphia *Medical and Surgical Reporter:*

The Municipal Council of Udine, in Northern Italy, has lately published a degree, in which it declares that, after having duly weighed and considered the advantages and drawbacks of cremation *versus* interment, it has come to the conclusion that the former is in every respect preferable, for the following reasons: 1. In a hygienic point of view, it is undoubtedly the best way of disposing of dead bodies. 2. It is a mark of progress, because, by making cremation optional, the individual is at liberty to choose between the two modes of burial. 3. Considered from a scientific, social, religious and sentimental point of view, no valid reasons can be brought forward against it, while many very good reasons might be quoted for it. 4. The expenses would not be heavier than those of an ordinary burial. Cremation has been long introduced, and is carried out, at Milan, as at Gotha. It is now also officially authorized in Paris. We shall be glad to welcome it in Philadelphia.

